# Comparative Evaluation of the Efficacy, Safety, and Corneal Dynamics of Overnight Orthokeratology in Teenagers and Adults With Myopia: A Prospective, Open‐Label Study in Taiwan

**DOI:** 10.1155/joph/6043551

**Published:** 2026-09-03

**Authors:** Shiuh-Liang Hsu, Chien-Liang Wu, Maylee Huang, Po-Yen Lee, Li-Yi Chiu

**Affiliations:** ^1^ Department of Ophthalmology, Kaohsiung Medical University Chung-Ho Memorial Hospital, Kaohsiung, Taiwan, kmu.edu.tw; ^2^ Department of Ophthalmology, School of Medicine, College of Medicine, Kaohsiung Medical University, Kaohsiung, Taiwan, kmu.edu.tw; ^3^ Department of Ophthalmology, Kaohsiung Medical University Gangshan Hospital, Kaohsiung, Taiwan; ^4^ Department of Ophthalmology, Taipei Municipal Wan Fang Hospital, Taipei Medical University, Taipei, Taiwan, tmu.edu.tw; ^5^ Optact International Co., Ltd, Taipei, Taiwan; ^6^ Graduate Institute of Clinical Medicine, Kaohsiung Medical University, Kaohsiung, Taiwan, kmu.edu.tw

**Keywords:** adult, children, myopia, orthokeratology, Taiwan

## Abstract

**Background:**

Myopia is a global public health concern, and its prevalence has been increasing year by year worldwide. Orthokeratology uses gas‐permeable contact lenses to reshape the corneal contour and correct low‐to‐moderate myopia. Orthokeratology has also been shown to reduce myopia progression in children. For adults, ortho‐K lenses remain a viable option for myopia correction; however, their usage rate is relatively low due to maintenance difficulties and safety concerns. To date, few studies have compared the efficacy, safety, and corneal dynamics of ortho‐K use between teenagers and adults.

**Methods:**

Subjects with myopia of either sex aged between 12 and 65 years were enrolled in this prospective open‐label study. The subjects were fitted with Progressive Boston Orthokeratology Shaping Lenses for overnight wear and were instructed to wear (before going to sleep) the lenses for > 7 h each night and remove them in the morning, for 9 months. Manifest refraction spherical equivalent (MRSE) and uncorrected visual acuity (UCVA) were assessed.

**Results:**

Overall, 68 subjects (134 eyes) completed the study, and of these, 15 (22.06%) subjects were teenagers (≤ 19 years) and 53 (77.94%) subjects were adults (> 19 years). In the teenager and adult groups, the mean MRSE changed from −2.84 D and −3.31 D at baseline to approximately 0.0 D in both groups at 9 months. UCVA improved significantly in both groups, reaching approximately Snellen 20/20 (logMAR 0) within the first few weeks of treatment and remaining stable throughout the study period. No clinically significant increases in intraocular pressure were observed during treatment, and endothelial cell density remained clinically stable. Mild corneal staining occurred in a small number of examinations and resolved with conservative management. After discontinuation, both MRSE and UCVA in both groups quickly returned to baseline by Week 4.

**Conclusion:**

These findings suggest that orthokeratology is an effective option for temporary correction of low‐to‐moderate myopia in both teenagers and adults. Within the limitations of the sample size, orthokeratology may be considered a safe and effective myopia correction option for adults.

## 1. Background

Myopia is a global public health concern, with its prevalence increasing year by year. According to a global trend study, the prevalence of myopia may reach 50% by 2050 [1]. Individuals with myopia have an increased risk of developing glaucoma, retinal detachment, and macular degeneration compared to individuals with emmetropia [2, 3].

Orthokeratology is an effective clinical technique that uses gas‐permeable contact lenses to temporarily reshape the cornea, primarily for the correction of low to moderate myopia [4–6]. These lenses are worn before sleep, kept in place overnight, and removed in the morning. Regular nighttime use reshapes the cornea, enabling clear vision during the day. Several studies have demonstrated that axial length elongation in children wearing ortho‐k lenses is reduced by 32%–55% compared with single‐vision spectacles or soft contact lenses [6–12].

In adults with myopia, axial length elongation may continue throughout life [13–15]. Therefore, orthokeratology may serve a dual purpose by providing effective vision correction while simultaneously contributing to myopia control. Recent research suggests that orthokeratology can enhance the quality of life in adults while posing minimal risk of adverse effects [16, 17]. However, it is unclear whether age has an impact on the speed and extent of myopia reduction using ortho‐k. Only one retrospective case study has compared the efficacy, predictability, and safety of long‐term orthokeratology, demonstrating that it is a safe and predictable procedure for both children and adults, with minimal adverse effects [18].

Therefore, the aim of this prospective study was to evaluate the efficacy and safety of overnight orthokeratology in individuals with mild‐to‐moderate myopia and to describe the temporal changes in refractive error, visual acuity, and corneal parameters during treatment.

## 2. Methods

### 2.1. Study Population

All subjects had an initial ocular examination to determine the eligibility. The criteria for enrollment in the study were as follows: either sex aged between 12 and 65 years (inclusive), best corrected visual acuity (BCVA) of ≥ 20/20 (LogMAR 0), refractive spherical correction of between −1.00 and −4.00 D, refractive cylindrical correction of between 0.00 and 1.50 D, flat K reading between 40.00 and 46.00 D, and healthy cornea as assessed by study investigator. Subjects with ocular or systemic disorders that would normally contraindicate contact lens wear, anterior eye clinical signs, use of topical ocular medication, pregnant, lactating, or planning pregnancy during the study period, severe dry eye (Schirmer Test, < 5 mm/5 min), pupil diameter > 5.0 mm, and those with an endothelial cell density (ECD) < 2000/mm^2^ were excluded from the study.

### 2.2. Study Design

This was a prospective open‐label study conducted between August 2009 and September 2010 at two major teaching hospitals (Kaohsiung Chung‐Ho Memorial Hospital and Taipei Municipal Wan Fang Hospital) in Taiwan. A total of 77 subjects were enrolled through advertisements placed in the hospitals, subjects attending the clinics at study site, and through direct interactions for participation. The subjects were fitted with Progressive Boston Orthokeratology Shaping Lenses for overnight wear made from Boston Equalens II material with DK of 127 (Bausch & Lomb, USA) and manufactured by Progressive Vision Technology (Dallas, TX, USA). Subjects were instructed to wear (before going to sleep) the lenses for > 7 h each night and remove it in the morning, for 9 months. Follow‐up examinations were scheduled after 1 h of awakening and about 6 h later at Day 2, Weeks 1 and 2, and Months 1, 2, 3, 6, and 9. Additional visits were scheduled whenever necessary for symptomatic reports of discomfort, change in vision, or damage or loss of lenses. When necessary, the lens prescription was changed for a better fit.

Corneal topography was obtained at each follow‐up visit to ensure proper fitting and corneal integrity. Standard subjective refraction techniques were used to determine the manifest refractive error, and results were converted to manifest refraction spherical equivalent (MRSE) for the ease of analysis. All subjects had undergone a comprehensive eye examination, including subjective refraction, uncorrected and corrected visual acuity, autorefraction, autokeratometry, corneal topography, intraocular pressure (IOP), ECD, and slit lamp examination at baseline and all subsequent follow‐up visits. Subjects discontinued the lens wear after 9 months and were examined at day 2, week 1 and month 1 to check the recovery to baseline visual acuity, refraction, and corneal shape. Data are presented as mean (standard deviation [SD]).

### 2.3. Statistical Analysis

The subjects were divided into two age groups: teenagers (≤ 19 years) and adults (> 19 years). Decimal visual acuity values were converted to logMAR using −log10 (decimal visual acuity) for statistical analysis. Data for refractive change was calculated from baseline to the last follow‐up visit at month 9. Changes in MRSE, uncorrected visual acuity (UCVA), IOP, and ECD from baseline to Month 9 were analyzed using paired Student’s *t*‐tests. Between‐group comparisons of continuous baseline variables were performed using Welch’s *t*‐tests, and sex was compared using Fisher’s exact test. A two‐tailed probability of 0.05 or less was considered statistically significant.

## 3. Results

**FIGURE 1:**
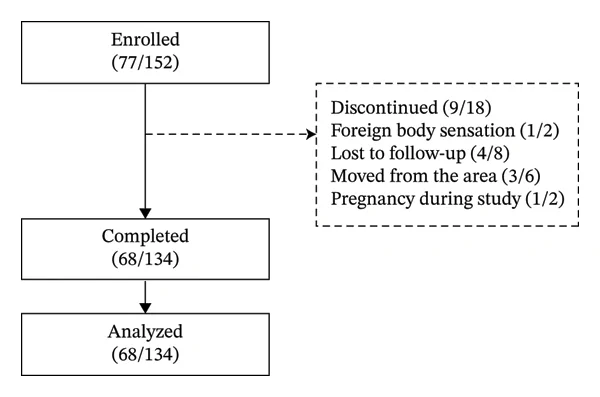
Flow diagram of participant enrollment, discontinuation, study completion, and analysis. A total of 77 participants (152 eyes) were enrolled. Nine participants (18 eyes) discontinued the study because of foreign‐body sensation (1 participant, 2 eyes), loss to follow‐up (4 participants, 8 eyes), relocation from the study area (3 participants, 6 eyes), or pregnancy during the study (1 participant, 2 eyes). Ultimately, 68 participants (134 eyes) completed the study and were included in the analysis.

A total of 77 subjects (152 eyes) were enrolled in this prospective study. Of these, 9 subjects discontinued participation before completing the study, including 2 teenagers and 7 adults, corresponding to attrition rates of 11.8% and 11.7%, respectively. Ultimately, 68 subjects (134 eyes) completed the 9‐month follow‐up period (Figure 1).

Among the 68 subjects who completed the study, 66 were enrolled bilaterally, whereas 2 subjects (1 teenager and 1 adult) were enrolled unilaterally. Of the 68 participants who completed the study, 53 (77.9%) were female. The overall mean age of the study population was 26.31 ± 9.18 years (range, 12–54 years). Based on age classification, 15 subjects (22.1%) were categorized as teenagers (≤ 19 years) and 53 (77.9%) as adults (> 19 years) (Table 1).

**TABLE 1:** Demographic and baseline ocular characteristics.

Characteristic	Teenagers ≤ 19 years (*n* = 15 subjects; 29 eyes)	Adults > 19 years (*n* = 53 subjects; 105 eyes)	*p* value
Age (years)	15.73 ± 2.28	29.30 ± 8.11	< 0.001
Sex, *n* (%)			0.079
Male	6 (40.0)	9 (17.0)	
Female	9 (60.0)	44 (83.0)	
MRSE, D	−2.84 ± 0.91	−3.31 ± 0.95	0.017
Myopia, D	−2.59 ± 0.91	−2.99 ± 0.93	0.042
Refractive astigmatism, D	−0.50 ± 0.47	−0.65 ± 0.49	0.149
Corneal astigmatism, D	−0.83 ± 0.65	−1.19 ± 0.58	0.011
UCVA, logMAR	0.90 ± 0.29	1.01 ± 0.29	0.079
IOP (mmHg)	14.84 ± 2.82	12.89 ± 2.92	0.002
ECD (cells/mm^2^)	3121.53 ± 195.43	3073.75 ± 353.10	0.342

*Note:* Data are presented as mean ± SD unless otherwise indicated. Age and sex were analyzed at the subject level; ocular characteristics were analyzed at the eye level. *p* values were calculated using Welch’s *t* test for continuous variables and Fisher’s exact test for sex. UCVA values were converted from decimal notation to logMAR using −log10 (decimal visual acuity). IOP, intraocular pressure; UCVA, uncorrected visual acuity.

Abbreviations: ECD, endothelial cell density; MRSE, manifest refraction spherical equivalent.

Significant baseline differences between the two groups were observed in age, MRSE, myopia, corneal astigmatism, and IOP. The overall baseline mean MRSE was −3.21 ± 0.96 D, and the mean UCVA was 0.99 ± 0.29 logMAR. The mean IOP was 13.31 ± 3.00 mmHg, and the mean ECD was 3084.09 ± 325.46 cells/mm^2^.

**FIGURE 2:**
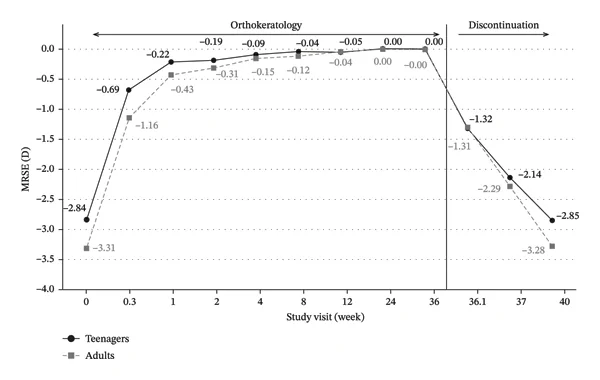
Changes in manifest refraction spherical equivalent during orthokeratology treatment and after discontinuation. Mean manifest refraction spherical equivalent (MRSE) values are shown for teenagers aged ≤ 19 years (29 eyes; solid line with circles) and adults aged > 19 years (105 eyes; dashed line with squares) during 36 weeks of orthokeratology treatment and four weeks after treatment discontinuation. MRSE rapidly approached plano after initiation of treatment, remained stable throughout the treatment period, and returned toward baseline after discontinuation. Values are expressed in diopters.

Orthokeratology treatment resulted in rapid improvement in refractive error and visual acuity. The mean MRSE decreased from −2.84 ± 0.91 D and −3.31 ± 0.95 D at baseline to approximately plano in both groups after 9 months of treatment (Table 2 and Figure 2). Paired *t*‐test analysis demonstrated statistically significant reductions in MRSE in both groups (*p* < 0.001). Following discontinuation of orthokeratology lens wear, MRSE gradually returned toward baseline levels within 4 weeks.

**TABLE 2:** Changes in ocular parameters after 9 months of orthokeratology.

Outcome	Group	Baseline	Month 9	Change from baseline	*p* value
MRSE, D	Teenagers (*n* = 29 eyes)	−2.84 ± 0.91	0.00 ± 0.00	2.84 ± 0.91	< 0.001
MRSE, D	Adults (*n* = 105 eyes)	−3.31 ± 0.95	0.00 ± 0.03	3.31 ± 0.95	< 0.001
UCVA, logMAR	Teenagers (*n* = 29 eyes)	0.90 ± 0.29	−0.02 ± 0.03	−0.92 ± 0.28	< 0.001
UCVA, logMAR	Adults (*n* = 105 eyes)	1.01 ± 0.29	−0.02 ± 0.04	−1.03 ± 0.28	< 0.001
IOP (mmHg)	Teenagers (*n* = 29 eyes)	14.84 ± 2.82	12.71 ± 2.50	−2.14 ± 2.17	< 0.001
IOP (mmHg)	Adults (*n* = 105 eyes)	12.89 ± 2.92	11.90 ± 2.66	−0.99 ± 2.22	< 0.001
ECD (cells/mm^2^)	Teenagers (*n* = 29 eyes)	3121.53 ± 195.43	3118.06 ± 185.37	−3.48 ± 109.82	0.866
ECD (cells/mm^2^)	Adults (*n* = 105 eyes)	3073.75 ± 353.10	3066.13 ± 347.40	−7.62 ± 35.02	0.028

*Note:* Data are presented as mean ± SD and were analyzed at the eye level. Change from baseline was calculated as the Month‐9 value minus the baseline value. *p* values were calculated using paired *t* tests. UCVA values were converted from decimal notation to logMAR using −log10 (decimal visual acuity). One teenager and one adult contributed only one eye to the analysis. IOP, intraocular pressure; UCVA, uncorrected visual acuity.

Abbreviations: ECD, endothelial cell density; MRSE, manifest refraction spherical equivalent.

**FIGURE 3:**
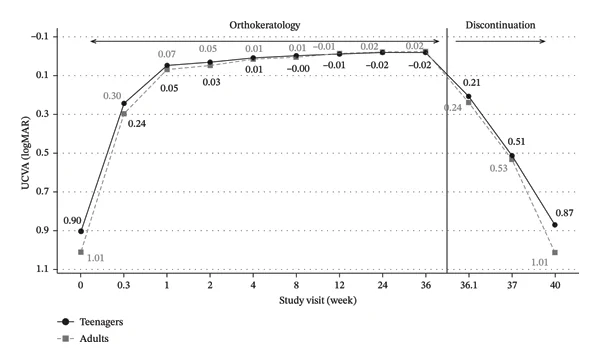
Changes in uncorrected visual acuity during orthokeratology treatment and after discontinuation. Mean uncorrected visual acuity (UCVA), expressed in logarithm of the minimum angle of resolution (logMAR) units, is shown for teenagers aged ≤ 19 years (29 eyes; solid line with circles) and adults aged > 19 years (105 eyes; dashed line with squares) during 36 weeks of orthokeratology treatment and four weeks after treatment discontinuation. UCVA improved rapidly after treatment initiation, remained stable during treatment, and returned toward baseline after discontinuation. The vertical axis is inverted so that better visual acuity appears higher in the figure.

Similarly, UCVA improved markedly after initiation of orthokeratology treatment. Most subjects achieved approximately 20/20 visual acuity (logMAR 0) within the first few weeks of treatment (Figure 3), and this level of visual acuity was maintained throughout the study period. Mean UCVA improved from 0.90 ± 0.29 to −0.02 ± 0.03 logMAR in teenagers and from 1.01 ± 0.29 to −0.02 ± 0.04 logMAR in adults. Paired *t*‐test analysis confirmed significant improvement in UCVA from baseline to Month 9 in both groups (both *p* < 0.001).

With regard to safety outcomes, small but statistically significant reductions in IOP were observed in both groups (both *p* < 0.001); however, all values remained within the normal physiological range. ECD remained clinically stable. No significant change was observed in teenagers (*p* = 0.866), whereas adults showed a small reduction of 7.62 ± 35.02 cells/mm^2^ (*p* = 0.028), which was not considered clinically meaningful.

Corneal astigmatism showed minimal change during the study period. The mean corneal astigmatism changed from −1.11 ± 0.62 D at baseline to −1.14 ± 0.73 D at Month 9, and the difference was not statistically significant.

## 4. Discussion

This prospective open‐label study evaluated the efficacy and safety of overnight orthokeratology in individuals with mild‐to‐moderate myopia across a broad age range. The results demonstrate that orthokeratology effectively reduced refractive error and improved visual acuity over the 9‐month treatment period, with no clinically significant adverse ocular events observed.

Orthokeratology has been widely studied as a strategy for myopia control in children. However, comparatively fewer studies have evaluated its clinical performance in older populations. Our findings indicate that orthokeratology can provide effective refractive correction in both teenagers and adults with low‐to‐moderate myopia.

In the present study, both MRSE and UCVA improved rapidly after initiation of orthokeratology treatment. Most participants achieved 20/20 vision (logMAR 0) within the early phase of treatment, and the visual outcomes were maintained throughout the study period. These findings are consistent with previous reports demonstrating the effectiveness of orthokeratology in correcting myopic refractive error and improving unaided visual acuity.

Following discontinuation of orthokeratology lens wear, refractive error and visual acuity gradually returned toward baseline values within 4 weeks. This reversibility is consistent with the temporary corneal reshaping mechanism of orthokeratology and has been reported in earlier studies.

In terms of safety, no clinically significant ocular complications were observed during the 9‐month treatment period. Although a small reduction in IOP was noted, all values remained within the normal physiological range and were not considered clinically meaningful. Furthermore, ECD remained clinically stable throughout the study period, suggesting that orthokeratology did not adversely affect corneal endothelial health. These findings are consistent with previous reports indicating that orthokeratology does not meaningfully alter ECD or morphology.

### 4.1. Limitation

Despite these encouraging findings, several limitations should be acknowledged. First, a formal a priori sample size calculation was not performed, and the sample size was determined by the number of eligible participants during the study period. Second, the distribution of subjects between teenagers and adults was uneven, which may limit the ability to draw definitive conclusions regarding potential age‐related differences. Larger prospective studies with balanced group sizes are warranted to further confirm these findings.

## 5. Conclusion

In conclusion, orthokeratology appears to be an effective and safe option for temporary myopia correction in individuals with mild‐to‐moderate myopia. The treatment produced rapid improvements in refractive error and visual acuity while maintaining a favorable safety profile during the 9‐month observation period.

## Author Contributions

Dr. Shiuh‐Liang Hsu and Dr. Chien‐Liang Wu participated in the design of the study, data collection, statistical analysis, and interpretation of the data. Dr. Maylee Huang participated in the design of the study and performed the statistical analysis. All authors participated in the manuscript development.

## Funding

This study was funded by Optact International Co., Ltd, Taipei, Taiwan.

## Disclosure

All authors have read and approved the final manuscript for submission.

## Ethics Statement

The study was conducted in accordance with International Conference on Harmonization Good Clinical Practices and principles that have their origin in Declaration of Helsinki. The study protocol was reviewed and approved by independent ethics committee or institutional review boards of Taiwan FDA and of each participating center. All study subjects provided written informed consent before any study related procedures. Written informed assents were obtained from subjects aged below 20 years in addition to written informed consent from their parents or guardians.

## Consent

All authors have contributed significantly and are in agreement with the content of the manuscript. All authors have signed the consent to publication.

## Conflicts of Interest

Dr. Maylee Huang is an employee of Optact International Co., Ltd, Taipei, Taiwan. The remaining authors declare no conflicts of interest.

## Data Availability

The datasets used and/or analyzed during the current study available from the corresponding author upon reasonable request.
